# Servicing open-source large language models for oncology

**DOI:** 10.1093/oncolo/oyae264

**Published:** 2024-09-24

**Authors:** Partha Pratim Ray

**Affiliations:** Department of Computer Applications, Sikkim University, Gangtok, Sikkim, India

I read with great interest the recent article by Iannantuono et al^1^ comparing large language models (LLMs) in answering immuno-oncology (IO) questions. The authors present a timely and important investigation into the potential utility of LLMs like ChatGPT and Google Bard in the rapidly evolving field of IO. Their findings highlight both the promise and limitations of these AI technologies for providing information to health care professionals and patients.

However, the authors rightly emphasize the risk of inaccurate or incomplete responses from LLMs, even from the best-performing models.^2-4^ This underscores the critical need for expert verification of LLM outputs before any clinical application. Importantly, the proprietary nature of the training data and algorithms used by commercial LLMs precludes full transparency and reproducibility.^5,6^

To address these limitations, I believe the medical AI community should prioritize the development and validation of open-source LLMs specifically trained on high-quality biomedical data. Models like BioGPT (https://github.com/microsoft/BioGPT), built on publicly available research literature, represent a promising direction. By focusing on well-defined medical tasks and enabling full scrutiny of the training process, such initiatives could yield LLMs that are more trustworthy and tailored to the unique demands of health care.^7^

The future of oncology is increasingly intertwined with the development of powerful generative AI technologies like open-source LLMs. By harnessing their vast potential through carefully curated, domain-specific training data and robust human oversight, we stand at the threshold of a new era in cancer care. Open-source LLMs, built upon the principles of transparency, reproducibility, and collaboration, hold the key to unlocking unprecedented insights from the ever-expanding corpus of oncology research and clinical data. These models, tailored to the unique challenges and complexities of cancer biology and treatment, will serve as indispensable allies in driving transformative advances in oncology.
